# S-1 plus cisplatin versus fluorouracil plus cisplatin in advanced gastric or gastro-esophageal junction adenocarcinoma patients: a pilot study

**DOI:** 10.18632/oncotarget.5959

**Published:** 2015-10-02

**Authors:** Yuhong Li, Miaozhen Qiu, Jianming Xu, Guoping Sun, Huishan Lu, Yunpeng Liu, Meizuo Zhong, Helong Zhang, Shiying Yu, Wei Li, Xiaohua Hu, Jiejun Wang, Ying Cheng, Juntian Zhou, Zengqing Guo, Zhongzhen Guan, Ruihua Xu

**Affiliations:** ^1^ Department of Medical Oncology, Sun Yat-Sen University Cancer Center, State Key Laboratory of Oncology in South China, Collaborative Innovation Center for Cancer Medicine, Guangzhou, China; ^2^ Department of Oncology, The Sidney Kimmel Comprehensive Cancer Center, The Johns Hopkins University School of Medicine, Baltimore, MD, USA; ^3^ Department of Oncology, 307 Hospital of the People's Liberation Army, Beijing, China; ^4^ Department of Oncology, The First Affiliated Hospital of AnHui Medical University, Hefei, China; ^5^ Department of General Surgery, Affiliated Union Hospital, Fujian Medical University, Fuzhou, China; ^6^ The Second Lab of Cancer Research Institute, The First Hospital of China Medical University, Shenyang, China; ^7^ Department of Oncology, Xiangya Hospital, Central South University, Changsha, Hunan, China; ^8^ Department of Oncology, Tangdu Hospital, The Fourth Military Medical University, Xi'an, China; ^9^ Department of Oncology, Tongji Cancer Center, Tongji Hospital, Tongji Medical College, Huazhong University of Science and Technology, Wuhan, China; ^10^ Stem Cell and Cancer Center, First Affiliated Hospital, Jilin University, Changchun, Jilin, China; ^11^ Department of Oncology, Affiliated Tumor Hospital of Guangxi Medical University, Nanning, China; ^12^ Department of Oncology, Changzheng Hospital, Shanghai, China; ^13^ Department of Oncology, Tumor Hospital of Jilin Province, Changchun, China; ^14^ Department of Oncology, Tumor Hospital of Hunan Province, Changsha, China; ^15^ Department of Oncology, Tumor Hospital of Fujian Province, Fuzhou, China

**Keywords:** gastric cancer, first-line chemotherapy, fluorouracil, S-1

## Abstract

The safety and efficacy of S-1 plus cisplatin in Chinese advanced gastric cancer patients in first line setting is unknown. In this pilot study, patients with advanced gastric or gastro-esophageal junction adenocarcinoma were enrolled and randomly assigned in a 1:1 ratio to receive S-1 plus cisplatin (CS group) or 5-FU plus cisplatin (CF group). The primary endpoint was time to progression (TTP). Secondary end points included overall survival (OS) and safety. This study was registered on ClinicalTrials. Gov, number NCT01198392. A total of 236 patients were enrolled. Median TTP was 5.51 months in CS group compared with 4.62 months in CF group [hazard ratio (HR) 1.028, 95% confidential interval (CI) 0.758-1.394, *p* = 0.859]. Median OS was 10.00 months and 10.46 months in CS and CF groups (HR 1.046, 95%CI 0.709-1.543, *p* = 0.820), respectively. The most common adverse events in both groups were anemia, leukopenia, neutropenia, nausea, thrombocytopenia, vomiting, anorexia and diarrhea. We find that S-1 plus cisplatin is an effective and tolerable option for advanced gastric or gastro-esophageal junction adenocarcinoma patients in China.

## INTRODUCTION

The incidence rate of gastric carcinoma varies dramatically worldwide and it is particularly high in Eastern Asia, especially in China [1]. Advanced gastric cancer (AGC) patients account for 40% of new Chinese gastric cancer patients and have a worse prognosis than that with early stage diseases [2]. Though first-line chemotherapy for AGC patients prolongs overall survival (OS) and improves quality of life (QoL) compared with best supportive care (BSC); the median survival for AGC patients who receive palliative chemotherapy is approximately 7 to 11 months [2-8].

The available cytotoxic agents for treatment of AGC patients included cisplatin, fluorouracil, oxaliplatin, irinotecan and taxane [3, 9-12]. S-1 is one of the oral fluoropyrimidines, consisting of tegafur, 5-chloro-2, 4-dihydropyrimidine, and potassium oxonate [13, 14]. Data from two phase II studies of single agent S-1 showed a response rate of 45% and 2-year survival of 17%, in association with 5% or lower frequencies of grade 3 or 4 toxic effects [15, 16].

Ajani JA et al. compared cisplatin/S-1 (CS) and cisplatin/5-fluorouracil (CF) in the first-line chemotherapy of AGC in a non-inferiority setting (FLAGS) and they found that CS was non-inferior to CF with a lesser toxicity profile [17]. However, the ‘lesser toxicity' observed in this trial was arguable, as a lower dose of cisplatin was used in CS group, and actually this trial failed to gain regulatory approval of S-1 in USA.

For any new agent, confirmation that efficacy and toxicity profiles are similar in non-Chinese and Chinese patients is important, given evidence of differences in treatment effects between populations with some agents (e.g. the gefitinib, which has a different toxicity profile in Asian and Western populations [18]).

Besides, Jin M et al. used S-1 monotherapy as second-line chemotherapy in AGC patients who had previously treated with cisplatin/ infusional fluorouracil and they found that S-1 monotherapy provided mild response rate and overall survival (OS) as well as favorable toxicity profile in the second-line setting for AGC patients [19].

In this study, we compared the effect of CS and CF in the first-line treatment for Chinese AGC patients, using the domestic agent of S-1 (produced by Shenzhen Wanle Pharmacy Company). This study was designed in 2008, before the publication of FLAGS study.

## RESULTS

### Patient characteristics

**Figure 1:**
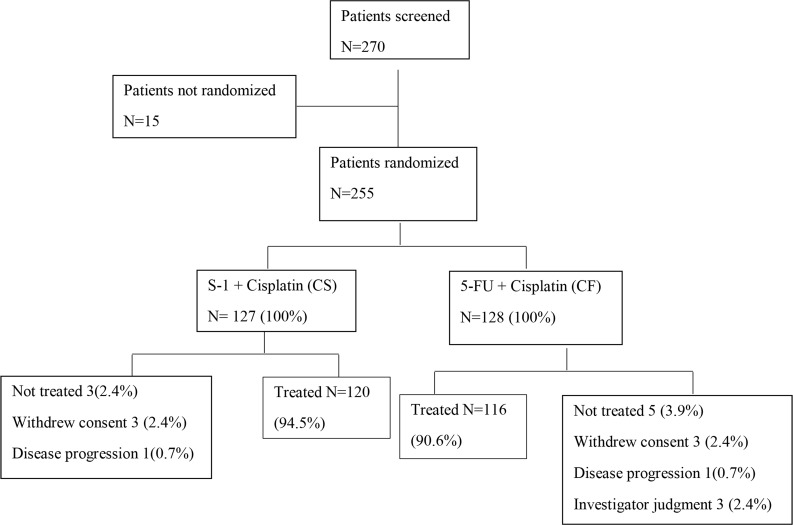
CONSORT diagram

Between October 2008 and June 2011, 255 patients were randomly assigned to either CS or CF group at 15 centers in China. The observation profile was listed in Figure 1.

Of the 255 patients, 236 was allocated to receive at least one cycle of CS (*n* = 120) or CF (*n* = 116). Table 1 showed demographics and baseline characteristics of patients included in the analysis. The basic features were well balance between these two groups. About 50% of the patients had low differentiated cancer. Approximately 85% of the patients had more than one site of metastasis and over half of the patients received previous gastrectomy.

**Table 1:** Baseline characteristics

Features	S1+Cisplatin (%)	5Fu+Cisplatin (%)	*P* value
Age			
Mean±SD	53.27±12.14	55.33±11.16	0.177
Gender			
Male	84 (70.0)	85 (73.3)	
Female	36 (30.0)	31 (26.7)	0.577
Race			
Han	116 (96.7)	111 (95.7)	
Others	4 (3.3)	5 (4.3)	0.695
Histology			
Low differentiated	57 (47.5)	65 (56.0)	
Moderate differentiated	28 (23.3)	17 (14.7)	
High differentiated	2 (1.7)	5 (4.3)	
Others	33 (27.5)	29 (25.0)	0.190
Location			
Stomach	70(58.33%)	73(62.93%)	
GE junction	22(18.33%)	10(8.62%)	
Stomach and GE junction	28(23.33%)	33(28.45%)	0.086
Sites of metastasis			
1	18(15.00%)	18(15.52%)	
>1	102(85.00%)	98(84.48%)	0.912
Previous gastrectomy			
Yes	65(54.17%)	64(55.17%)	
No	55(45.83%)	52(44.83%)	0.877
PS			
0	28(23.33%)	29(25.00%)	
1	85(70.83%)	83(71.55%)	
2	7(5.83%)	4(3.45%)	0.673

SD: Standard deviation; GE: Gastroesophageal; PS: Performance status

### Efficacy

The mean dose intensity of S-1 and Fluorouracil was 278.46 mg/m^2^/week and 964.48mg/m^2^/week respectively. The median cycles and duration of treatment in these two groups were 3.53 and 3.46 (*p* = 0.78), 4.03 and 2.87 months (*p* = 0.0002), respectively.

Median time to progression (TTP) in CS and CF groups was 5.51 months [95% confidence interval (CI): 4.59-6.26] versus 4.62 months (95% CI: 4.00-6.33), hazard ratio (HR) 1.028, 95% CI 0.76-1.39, *p* = 0.86, Figure 2. There was no difference of median TTP among the three stratification factors (Table 2). The response rate was 22.5% [27 patients got a partial response (PR)] in CS group and 21.6% in CF group [2 complete response (CR) and 23 PR], *p* = 0.86. The OS between these two groups was showed in Figure 3. The median OS for patients in CS and CF group was 10.00 months(95% CI: 8.59-14.52) and 10.46 months (95% CI: 8.92-13.84), *p* = 0.82.

**Table 2:** Stratification factors

			Number (%)	*P* *value	mTTP (months)	95% CI	*P*^#^ value
PS	0	CS group	28(23.33%)	0.57	5.54	4.33-8.16	0.87
		CF group	29(25.00%)		4.20	2.75-10.46	
	1	CS group	85(70.83%)		5.64	4.20-6.30	0.64
		CF group	83(71.55%)		5.05	4.00-6.56	
	2	CS group	7(5.84%)		4.41	3.39-5.62	0.84
		CF group	4(3.45%)		4.61	3.36-5.20	
Numbers of metastasis	1	CS group	18(15.00%)	0.91	6.26	3.61-8.52	0.2
		CF group	18(15.52%)		5.64	4.00-6.26	
	>1	CS group	102(85.00%)		5.25	4.33-6.26	0.63
		CF group	98(84.48%)		4.33	3.90-5.87	
Gastrectomy	No	CS group	65(54.17%)	0.88	5.25	4.23-6.26	0.86
		CF group	64(55.17%)		4.20	3.90-6.10	
	Yes	CS group	55(45.83%)		5.77	4.33-7.57	0.95
		CF group	52(44.83%)		5.05	3.70-9.97	

TTP: Time to progression

CS: cisplatin and S-1; CF: cisplatin and fluorouracil

* The comparison of patients in different stratification factors

# The comparison of TTP for patients in CS and CF groups.

**Figure 2:**
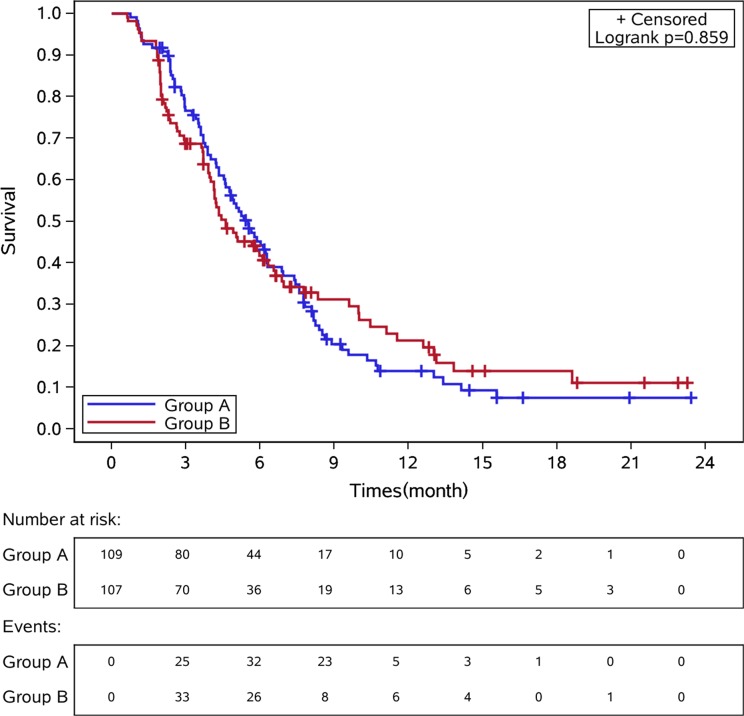
Kaplan-Meier curves of time to progression in these two groups of patients Group A: CS group. Group B: CF group.

### Safety

No significant difference in the overall rate of adverse events between these two groups was detected (all grades, Table 3). Anemia, neutropenia, nausea, thrombocytopenia, vomiting and anorexia were the most frequently reported adverse events. Patients assigned to CS group had slightly higher rates of thrombocytopenia, abdominal pain, hyperbilirubinemia, pigmentation and stomatitis than did patients assigned to CF group.

Patients in CS group had higher incidence rate of grade 3 or 4 adverse events (Table 3). Serious adverse events (grade 3 or 4 adverse events) were reported in 73 (60%) patients in CS group and 47 (36%) patients in CF group, *p* = 0.0015. The most common grade 3 or 4 adverse events were neutropenia, anemia, leucopenia and thrombocytopenia in CF-treated patients.

The proportion of patients reporting an adverse event that led to dose modifications or interruptions did not differ between these two groups. No drug related death in both groups was reported in our study.

**Table 3:** Adverse event in our study compared with the FLAGS study

	S-1plus cisplatin				5-FU plus cisplatin			
	Our result (%)		FLAGS Study (%)		Our result (%)		FLAGS Study (%)	
	All	Grade 3/4	All	Grade 3/4	All	Grade 3/4	All	Grade 3/4
Neutropenia	68.6	36.4	28.6	18.6	55.9	13.6	47.2	40.0
Anemia	80.2	25.6	44.0	15.7	72.0	11.9	46.1	19.3
Leucopenia	71.9	17.6	17.5	7.3	62.7	5.9	23.0	13.8
Thrombocytopenia	44.6	14.1	17.7	5.4	26.3	4.2	22.8	8.5
Diarrhea	24.0	5.8	29.2	4.8	17.8	1.7	38.4	4.5
Vomiting	43.0	4.1	48.0	7.9	42.4	5.1	55.3	9.6
Nausea	50.4	3.3	61.6	7.5	60.2	4.2	67.3	9.6
Anorexia	38.0	2.5	31.5	6.0	41.5	3.4	34.8	5.5
Constipation	20.7	0	/	/	22.0	0.9	/	/
Abdominal pain	15.7	1.7	25.1	7.3	5.9	0	22.4	5.3
Fatigue	21.5	0	39.3	12.3	22.0	0	39.4	13.2
Abnormal pigmentation	10.7	0	/	/	1.7	0	/	/
Hypokalemia	4.1	0	6.9	3.6	4.2	0.9	16.7	10.8
Dehydration	/	/	12.1	4.8	/	/	15.6	7.5
Weight loss	0.8	0	28.4	4.0	0	0	32.3	6.1
Stomatitis	3.3	0.8	6.3	1.3	6.8	0.9	30.1	13.6

**Figure 3:**
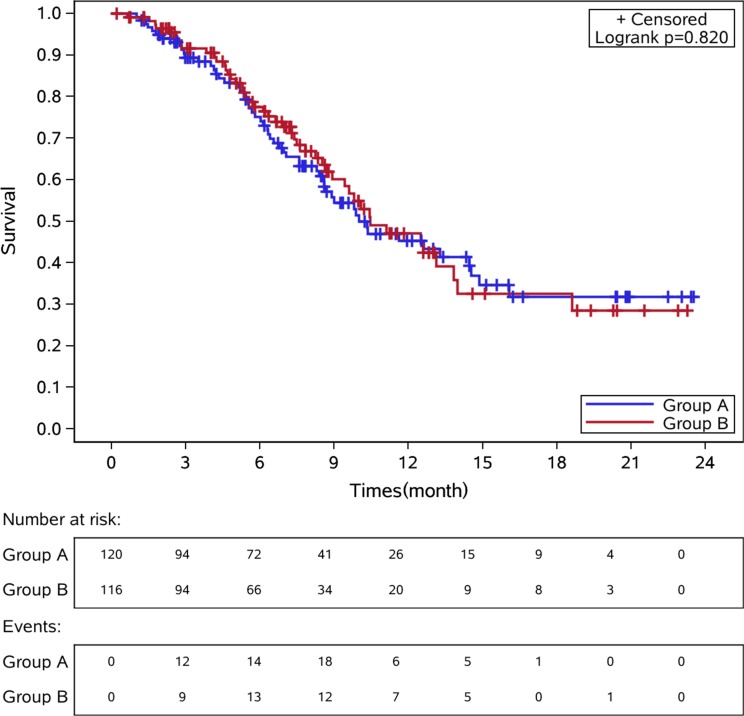
Kaplan-Meier curves of overall survival in these two groups of patients Group A: CS group. Group B: CF group.

## DISCUSSION

Our findings showed that CS had comparable treatment effect with CF, with respect to similar results of TTP, RR and OS. Patients assigned to S-1 had a 0.9 month longer TTP than those allocated to a continuous infusion of fluorouracil. These findings for S-1 were consistent with the previous reports [5, 15]. Drug development for gastric cancer has been focused on replacement of intravenous fluorouracil with oral agents [4, 20]. Taken together with our findings, S-1 can be an alternative option for continuous infusion of fluorouracil in China.

Toxic effects of S-1 have been reported to be more severe in individuals from USA than those from Asian, indicating different dosage recommended for these populations [13, 21]. Since similar discrepancies in toxic effects have been noted with tegafur and uracil, ethnic variations seem to be a factor with these dihydropyrimidine dehydrogenase inhibitory fluoropyrimidines [22]. In 2008, an American Society of Clinical Oncology (ASCO) abstract showed that S-1 plus cisplatin was superior to continuous infusion of fluorouracil plus cisplatin. Outside Asia, despite differences in dose and schedule of S-1 from Asian trials, S-1 plus cisplatin was associated with fewer toxic effects, slightly better survival, and non-inferiority compared with fluorouracil plus cisplatin [5]. In our study, no significant difference in the overall rate of adverse events between CS and CF groups was found. Despite a higher rate of grade 3 or 4 adverse events in CS group than in CF group, the number of patients discontinuing treatment due to toxicity was limited, and patients in CS group were able to stay on treatment longer than those in CF group. The most common grade 3 or 4 adverse events was hemorrhage. Early and effective prophylaxis as well as management of adverse events were helpful to ensure that patients would remain on the treatment.

The two treatment regimens were different in terms of cycle length: 5 weeks in CS group and 4 weeks in CF group. It seemed that the treatment duration was biased from the beginning favoring the experimental arm. However, patients in these two groups received the scans with the same frequency, every 8 weeks. This could eliminate the potential bias from different cycle lengths. There were different dose schedules for S1, including one week on one week off, two weeks on two weeks off, two weeks on one week off, four weeks on two weeks off and three weeks on two weeks off [5, 13, 15-17, 23-25]. There was no study to compare the difference among these different dose schedules in efficacy and safety. It is reasonable to assume that longer off time would produce less side effect and that is why we pick up the dose schedule of 3 weeks on and two weeks off. We confirmed that this dose schedule was tolerable in Chinese AGC patients.

Oxaliplatin which has potentially improved efficacy and tolerance for platinum-based chemotherapy in AGC patients, has been widely used in the treatment of advanced gastric cancer [4, 8, 26]. However, cisplatin combined with fluorouracil was considered as the standard treatment for control group, the aim of our study was to make a direct comparison between S-1 and fluorouracil, so we chose cisplatin in both groups while not oxaliplatin.

In conclusion, we found that S-1 plus cisplatin was comparable to fluorouracil plus cisplatin, with a similar TTP and tolerable toxicity. In China, central venous catheterization or peripherally inserted central catheterization (PICC) would be established for patients to receive continuous infusion of 5Fu, but unnecessary for administration of cisplatin. With the advantage of oral administration, patients in S-1 group avoided the venous port. Based on our finding, S-1 plus cisplatin, was an acceptable substitute for infusion 5-FU plus cisplatin and an appropriate option as first-line chemotherapy for advanced gastric cancer patients in China. This pilot study provides some basic information for the further phase III clinical study for S1 in China.

## MATERIALS AND METHODS

### Patient enrollment

During October 2008 and June 2011, we prospectively recruited AGC patients all over China. Patients were enrolled based on the following criteria: 1) Men or women, aging between 18 and 75 years old; 2) Histologically confirmed inoperable locally advanced, recurrent, or metastatic adenocarcinoma of the stomach or gastro-esophageal junction; 3) Eastern Cooperative Oncology Group (ECOG) performance status (PS) 0*-*2; 4) Adequate organ function and measurable diseases; 5) At least one measurable lesion with a diameter ≥ 20 mm using conventional Computed Tomography (CT) or Magnetic Resonance Imaging (MRI) scans or ≥ 10 mm using spiral CT scans; 6) A life expectancy of at least 3 months.

The exclusion criteria included: 1) Pregnant or lactating women or women of childbearing potential with positive pregnancy test at baseline. Postmenopausal women with amenorrhea for at least 12 months to be considered of non-childbearing potential; 2) No target lesion; 3) No CT evaluation; 4) Prior systemic therapy for advanced or metastatic disease (for instance, cytotoxic chemotherapy or history of another malignancy within the last five years except cured basal cell carcinoma of skin and cured carcinoma in-situ of uterine cervix. active/passive immunotherapy); 5) History or evidence upon physical examination of central nervous system diseases (for example, primary brain tumor, seizure not controlled with standard medical therapy, or any brain metastases); 6) Severe comorbidities including angina, intestinal obstruction, diarrhea and active gastrointestinal bleeding.

### Ethics statement

All patients provided written informed consent. Approvals for the study protocol (and any modifications thereof) were obtained from independent ethics committees. The study was undertaken in accordance with the ethical standards of the World Medical Association Declaration of Helsinki. This study was registered on ClinicalTrials. Gov, number NCT01198392.

### Treatments

Patients who satisfied all eligibility criteria were randomly assigned in a 1:1 ratio to receive S1 (Shenzhen Wanle Pharmaceutical Co., Ltd. China) plus cisplatin (CS group) or fluorouracil plus cisplatin (CF group). As a pilot study, there is no need for sample size calculation. We planned to enroll 270 patients.

Randomized grouping information for each patient was generated by central randomization system. At randomization, patients were stratified by ECOG PS (0-1 *vs*. 2), numbers of metastasis sites (1 *vs*. > 1) and gastrectomy (yes *vs* no). Neither patients nor investigators were masked to treatment assignment in this open-label study.

S-1 was given as 40mg/m^2^ twice daily on day 1-21 and cisplatin was 20mg/m^2^ ivdrip on day 1-4, repeated every 5 weeks in the CS group. In the CF group, 5-Fu was given as 800 mg/m^2^/d CI 120h, and the dosage of cisplatin was 20mg/m^2^ iv on day 1-4, repeated every 4 weeks. The treatment would continue until disease progression, unacceptable toxicity, and withdrawal of consent or 6 cycles. Infusion treatment would be administrated in the inpatient department. Chemotherapy dose adjustments were allowed. Crossover to the other group at the time of disease progression was not allowed.

The primary endpoint was time to progression (TTP), defined as time from randomization until disease progression (PD) or death from any cause. Secondary endpoint included overall survival (OS) and safety. Investigators assessed tumor response and progression every 8 weeks, either radiologically using Response Evaluation Criteria in Solid Tumors (RECIST) version 1.1 or clinically if a patient could not have radiological examination. Patients were followed up until death, loss to follow-up or end of study. Efficacy and safety data were monitored by an independent data monitoring committee. Adverse events were assessed according to the National Cancer Institute Common Terminology Criteria for Adverse Events (NCI-CTCAE) version 3.0 and serious adverse events according to International Conference on Harmonization guidelines.

### Treatment assessments

CT scans of the chest, abdomen and pelvic were performed for tumor assessment 2 weeks before and repeated repeated every 8 weeks during the treatment. After the treatment, patients were recommended to receive the CT scans every 3 months. All patients in this study had full information of follow-up. The last date of follow-up was June 1st, 2014.

### Statistical analysis

The intention-to-treat (ITT) principle was applied. All statistical analysis was performed by Statistical Package of Social Sciences 16.0 software. TTP was adjusted for stratification factors. Patient characteristics were described using summary statistics. *P* value for comparing patient characteristics was calculated using chi squared test. Statistical significance was set at two-sided *P* < 0.05. All efficacy analysis were done on the full population. No imputation was made for missing assessments. Safety analysis included patients who received at least one dose of study medication.
